# Antibacterial activity of a DNA topoisomerase I inhibitor versus fluoroquinolones in *Streptococcus pneumoniae*

**DOI:** 10.1371/journal.pone.0241780

**Published:** 2020-11-03

**Authors:** Myriam V. Valenzuela, Mirian Domenech, Patricia Mateos-Martínez, Fernando González-Camacho, Adela G. de la Campa, Maria Teresa García

**Affiliations:** 1 Departamento de Genética, Unidad de Microbiología, Fisiología y Microbiología, Universidad Complutense, Madrid, Spain; 2 Unidad de Neumococos, Centro Nacional de Microbiología, Instituto de Salud Carlos III, Madrid; Spain; 3 Unidad de Genética Bacteriana, Centro Nacional de Microbiología, Instituto de Salud Carlos III, Madrid; Spain; 4 Presidencia, Consejo Superior de Investigaciones Científicas, Madrid, Spain; Universita degli Studi della Campania Luigi Vanvitelli, ITALY

## Abstract

The DNA topoisomerase complement of *Streptococcus pneumoniae* is constituted by two type II enzymes (topoisomerase IV and gyrase), and a single type I enzyme (topoisomerase I). These enzymes maintain the DNA topology, which is essential for replication and transcription. While fluoroquinolones target the type II enzymes, seconeolitsine, a new antimicrobial agent, targets topoisomerase I. We compared for the first time the *in vitro* effect of inhibition of topoisomerase I by seconeolitsine and of the type II topoisomerases by the fluoroquinolones levofloxacin and moxifloxacin. We used three isogenic non-encapsulated strains and five non-vaccine serotypes isolates belonging to two circulating pneumococcal clones, ST63^8^ (2 strains) and ST156^9V^ (3 strains). Each group contained strains with diverse susceptibility to fluoroquinolones. Minimal inhibitory concentrations, killing curves and postantibiotic effects were determined. Seconeolitsine demonstrated the fastest and highest bactericidal activity against planktonic bacteria and biofilms. When fluoroquinolone-susceptible planktonic bacteria were considered, seconeolitsine induced postantibiotic effects (1.00−1.87 h) similar than levofloxacin (1.00−2.22 h), but longer than moxifloxacin (0.39−1.71 h). The same effect was observed in sessile bacteria forming biofilms. Seconeolitsine induced postantibiotic effects (0.84−2.31 h) that were similar to those of levofloxacin (0.99−3.32 h) but longer than those of moxifloxacin (0.89−1.91 h). The greatest effect was observed in the viability and adherence of bacteria in the postantibiotic phase. Seconeolitsine greatly reduced the thickness of the biofilms formed in comparison with fluoroquinolones: 2.91 ± 0.43 μm (seconeolitsine), 7.18 ± 0.58 μm (levofloxacin), 17.08 ± 1.02 μm (moxifloxacin). When fluoroquinolone-resistant bacteria were considered, postantibiotic effects induced by levofloxacin and moxifloxacin, but not by seconeolitsine, were shorter, decreasing up to 5-fold (levofloxacin) or 2-fold (moxifloxacin) in planktonic cells, and up to 1.7 (levofloxacin) or 1.4-fold (moxifloxacin) during biofilm formation. Therefore, topoisomerase I inhibitors could be an alternative for the treatment of pneumococcal diseases, including those caused by fluoroquinolone-resistant isolates.

## Introduction

*Streptococcus pneumoniae* is a main human pathogen. The implementation of the 7-valent and 13-valent conjugate vaccines, which include the serotype-specific polysaccharides more often associated with resistance to antibiotics, have led to a decline of invasive pneumococcal disease and penicillin resistance [1]. However, since vaccination only protects against 14–25% of serotypes, an increase in non-vaccine serotypes has been detected [2–4]. Non-encapsulated isolates, which are the etiological agents of 3–19% of pneumococcal diseases [5,6] and more prone to form biofilm than encapsulated strains [7], have also emerged.

*S*. *pneumoniae* colonizes the human nasopharynx and persists as an asymptomatic commensal [8]. The ability to produce biofilms is essential for this colonization [9] and for its dissemination to other body sites [10]. Dissemination leads to the development of sinusitis, conjunctivitis or acute otitis media and, eventually, invasive diseases, such as bacteremic pneumonia, meningitis and sepsis [11,12]. Pneumococcal biofilms are not only present in the nasopharynx, but also in adenoid and mucosal epithelial tissues in children with recurrent middle-ear infections and otitis media [13], sinus mucosa of patients with chronic rhinosinusitis [14], and in the lungs of mice infected with *S*. *pneumoniae* [15]. The clinical relevance of biofilms relies also on their capacity to act as a reservoir of antibiotic-resistant bacteria [16]. Biofilms are 1000-fold more tolerant and/or resistant to antibiotics than planktonic cells [17]. Thus *S*. *pneumoniae* biofilms are not effectively cleared during antimicrobial treatment [18]. In addition, biofilms have an inherent tolerance to host defenses [19]. Therefore, biofilm antibiotic therapy demands the use of higher doses of antibiotics over prolonged periods.

Treatment guidelines for pneumonia recommend respiratory fluoroquinolones (FQs) [20,21], given their broad spectrum, low resistant rate, excellent penetration into the bronchial mucosa and a pharmacokinetic profile that facilitates treatment with a single daily dose [22]. FQs target type II DNA topoisomerases: DNA topoisomerase IV (Topo IV: ParC_2_ParE_2_) and DNA gyrase (GyrA_2_GyrB_2_). *S*. *pneumoniae* has another DNA topoisomerase of type I (topoisomerase I, Topo I). These three topoisomerases maintain the DNA topology and solve topological problems associated with DNA transactions [23].

Resistance to FQs in *S*. *pneumoniae* is caused mainly by mutations that change amino acids of the quinolone resistance–determining regions of the subunits of Topo IV and of GyrA subunit of gyrase [24,25]. Although FQ-resistance in *S*. *pneumoniae* is maintained at low prevalence (< 3%) in Spain [26,27] and the rest of Europe [28], rates are higher in Canada (7.3%) [29] and in some locations of Asia (10.5%) [30]. An increase in FQ-resistance in *S*. *pneumoniae* would eventually occur if the FQ use is increased. Finding new antibiotics against *S*. *pneumoniae*, acting on new targets, is an important clinical need. Topo I has been proposed as a new antibacterial target [31]. Some alkaloids inhibited *in vitro* the enzymatic activity of *Escherichia coli* Topo I [32]. Our group has reported the inhibition of both Topo I activity and growth of *S*. *pneumoniae* [33] and *Mycobacterium tuberculosis* [34] by two new boldine-derived alkaloids, seconeolitsine (SCN) and N-methyl-seconeolitsine, without affecting human cell viability. The specific inhibition of Topo I by SCN *in vivo* is supported by the attenuation of growth inhibition under conditions of Topo I overproduction in *S*. *pneumoniae* [33], and by an increased susceptibility in a *M*. *tuberculosis* strain that has lower levels of Topo I [34]. In addition, SCN causes hyper-negative supercoiling in replicating plasmids in both species [33−35], consistent with the inhibition of Topo I, the main DNA-relaxing enzyme in these bacteria. This increase in supercoiling in *S*. *pneumoniae* triggers a coordinated global transcriptional response [35]. Therefore, Topo I could be considered a suitable new drug target, and boldine-derived alkaloids are attractive lead-compounds for further antibiotic development.

The aim of this study was to analyze the main DNA topoisomerases of *S*. *pneumoniae* as antibiotic targets by comparing the effects of inhibiting these enzymes in planktonic bacteria and biofilms. The antibacterial activity of SCN (Topo I inhibitor) was compared with that of two respiratory FQs, levofloxacin (LVX) and moxifloxacin (MXF), which inhibit the type II DNA topoisomerases. Minimal inhibitory concentrations (MICs), killing-curves and post antibiotic effects (PAEs) were tested and compared for the three compounds.

## Material and methods

### Bacterial strains

Eight *S*. *pneumoniae* strains were used (Table 1): 3 isogenic laboratory strains (R6, T1 and T2) that have been previously characterized [36], and two groups of clinical isolates belonging to two circulating pneumococcal clones (ST63^8^ and ST156^9V^). Isolates belonging to the ST63^8^ and ST156^9V^ clones were selected from our previous study [26]. Each group included strains with different susceptibility to ciprofloxacin (CIP) due to mutations in *parC* or both *parC* and *gyrA*.

**Table 1:** Characteristics of *S*. *pneumoniae* strains.

Group^a^	Strain^b^	Description	Residue change	MIC (μg/ml)^c^				Microbiological susceptibility categorization^d^		
				CIP	LVX	MXF	SCN	CIP	LVX	MXF
Laboratory strains	R6	Avirulent, uncapsulated	None	0.5	1	0.06	8	S	S	S
	T1	R6 derivative,	ParC S79F	4	1	0.06	4	LL-R	S	S
	T2	T1 derivative	ParC S79F	32	16	2	4	HL-R	HL-R	HL-R
			GyrA S81F							
ST63^8^ clone	2390	Blood isolate	ParC S79F	8	2	0.25	4	LL-R	S	S
	3498	Blood isolate	ParC S79F	32	16	4	4	HL-R	HL-R	HL-R
			GyrA S81F							
ST156^9V^ clone	3360	Eye isolate	None	1	0.5	0.06	4	S	S	S
	2194	Sputum isolate	ParC S79Y	4	1	0.12	4	LL-R	S	S
	1920	Bronchoalveolar isolate	ParC S79F	64	16	2	4	HL-R	HL-R	HL-R
			GyrA S81F							

^a^ Clone is named as sequencing type (ST) with the serotype in superscript.

^b^ Strains R6, T1 and T2 have been previously characterized [36]. The rest of isolates were selected from our previous studies [26].

^c^ MICs were determined by the microdilution method. The results are the average of three independent replicates.

^d^ S, susceptible. LL-R, low-level resistant: CIP MIC≥ 4 μg/ml, LVX MIC > 2 μg/ml; MXF MIC ≥ 1 μg/ml. HL-R, high-level resistance: CIP MIC ≥16 μg/ml, LVX MIC ≥ 16 μg/ml, MXF MIC ≥ 4 μg/ml.

### Susceptibility tests

MICs were determined by the microdilution method, based on the Clinical and Laboratory Standards Institute procedure [37]. Serial 2-fold dilutions of the antibiotics (between 64 and 0.03 μg/ml), were dispensed into 96-well polystyrene microtiter plates with bacteria cultures at a concentration of 10^5^ colony-forming units (CFU)/ml (200 μl final volume). Media used, named AGCH, was a casein hydrolysate-based medium with 0.3% sucrose and 0.2% yeast extract [38]. Plates were incubated at 37°C for 24 h in the presence of 5% CO_2_. The MIC was defined as the lowest concentration of drug without visible growth.

### Killing assays

The kinetics of killing of planktonic bacteria was studied in AGCH (a suitable medium for planktonic growth). Bacteria were treated with the drugs for 8 h by exposing 200 μl of about 10^4^ CFU/ml of R6 strain at sub-inhibitory (0, 1/8 ×, 1/4 ×, 1/2 × MIC) or inhibitory concentrations (1 ×, 2.5 ×, 5 ×, 10 × MIC) in 96-well polystyrene microtiter plates. Plates were incubated at 37°C with 5% CO_2_. Viable count was determined by plating 50 μl on Mueller-Hinton Blood Agar plates (Becton Dickinson). Biofilm formation was determined by the ability of cells to adhere to the walls and base of 96-well (flat-bottom) polystyrene microtiter plates (Costar 3595; Corning Incorporated), using a modification of a previously reported protocol [39]. R6 was grown to an OD_595_ = 0.5 in CpH8 medium (a suitable medium for the formation of biofilms) containing 33 mM potassium phosphate buffer at pH 8.0 [40]. Then, bacteria were diluted 1/100 and dispensed in the microtiter plates (200 μl per well). Plates were incubated at 34°C for 6 h to develop the biofilm. Then, cultures were rinsed with fresh media and concentrations equivalent to 5 × MIC of LVX, MXF or SCN were added and plates were incubated for 24 h at 34°C. Biofilm formation was analyzed by staining with crystal violet.

### Post antibiotic effects (PAE) in planktonic bacteria

PAE in planktonic bacteria was calculated by measuring bacteria re-growth after antibiotic treatment using the viable plate count method [41]. 10^8^ CFU/ml of bacteria in AGCH were treated for 1 h with different drug concentrations in 24-well (flat bottom) polystyrene microtiter dishes. The treatment was finished by a 1/1000 dilution with fresh media, and cultures were incubated for 6 h. Viable bacteria (CFUs) were counted every 2 h by plating 50 μl of culture on Mueller-Hinton blood agar plates. PAE was quantified with the formula PAE = T- C. It measures the time required for the viable bacteria counts to increase by 1 log_10_ above the counts observed immediately after washing in exposed cultures (T) regarding antibiotic unexposed cultures (C). PAE ≥ 0.5 h was considered significant [42]. Viability of R6 in PAE-phase was observed with a fluorescence microscopy OLYMPUS BXG1 every 2 h after the end of the treatment. A LIVE/DEAD BacLight bacterial viability kit L-7012 (Invitrogen-Molecular Probes) was used to monitor the viability of bacterial populations as a function of the membrane integrity of the bacteria: cells with a compromised membrane are considered to be damaged (stain yellow) or dead (stain red); whereas cells with an intact membrane will stain green [43]. BacLight-stained cells were observed under fluorescence microscopy (Olympus BXG1). At least 100 cells were analyzed for viability.

### PAE in biofilm

R6 and T2 strains were grown in CpH8 medium to OD_595_ = 0.5, diluted 1/100 and dispensed in 96-well flat-bottom polystyrene microtiter dishes. Plates were incubated at 34°C to get 10^6^ CFU/ml adhered to the walls base. Then, attached bacteria were treated with drugs during 1 h at 34°C. Drug treatment was finished by rinsing cultures three times with CpH8 and plates were incubated for 6 h. PAE was quantified by viable count method. At each time point, wells were washed three times with CpH8 and filled with phosphate-buffered saline to remove planktonic bacteria. The surfaces of the wells were vigorously scraped and the number of viable cells was estimated by plating 50 μl of culture on Mueller-Hinton Blood Agar. Bacteria in the biofilm was estimated by staining with crystal violet (50 μl of a 1% solution was added to each well), measuring *A*_595_ using a microplate absorbance reader 2020 (Anthos Labtec Instruments GmbH). Plates were then incubated at room temperature for 15 min, rinsed 3 times with 200 μl of water, and air dried. Stained biofilm was quantified by solubilizing it with 95% ethanol (200 μl/well) and determination of *A*_595_. Structure of biofilms was also observed by confocal laser scanning microscopy. Bacteria were grown on glass-bottom dishes (WillCo-dish; WillCo Wells B. V., The Netherlands) in 2 ml of CpH8 medium for 1.5 h at 34°C to get a biofilm with 10^6^ CFU/ml of viable bacteria. Culture medium containing planktonic bacteria was removed, and the biofilms were incubated in CpH8 with the drug for 1 h at 34°C. Then, antibiotic was rinsed 3 times with CpH8. Controls were run with the same amount of CpH8 without antibiotics. Biofilms were also stained with the bacterial viability *Bac*Light kit (Invitrogen^TM^, Thermo Fisher Scientific), showing viable (green fluorescence) and non-viable (red fluorescence) bacteria. After staining, they were rinsed with 0.5 ml of phosphate-buffered saline. Observations were made using a Leica TCS-SP2-AOBS (Acousto Optical Beam Splitter)-UV (Ultraviolet) or Leica TCS-SP5-AOBS confocal microscope (Mannheim, Germany) with objective HPX PL APO CS 63X/1.4 oil immersion and zoom 2. Laser line at 488nm for excitation of SYTO9, and Alexa fluor-488 were provided by an Argon laser and laser line 561nm for excitation of Propidium iodide (PI) a DiodeP Solid State laser. Detection ranges were set to eliminate crosstalk between fluorophores. The image resolution was 8 bits and format 512 × 512 pixels. Laser intensity and gain were kept the same for all images. Images were analyzed using LCS software from LEICA. Maximum intensity projections were obtained in the *x*–*y* (individual scans at 0.5–1 μm intervals) and *x*–*z* (images at 5–6 μm intervals) planes.

### Statistical analysis

Data for the assays include the mean ± standard error of at least two independent experiments. For multiples comparisons, one-way analyses of variance (ANOVA) with 95% significance level were performed. The *Statgraphics Statgraphics Centurion XVI* statistical packge was used for all analyses. Differences were considered statistically significant when *P* <0.05.

## Results

### SCN shows higher and faster bactericidal activity than FQs against *S*. *pneumoniae* R6

MICs of three FQs and SCN against eight bacterial strains were determined (Table 1). The strains tested include three isogenic non-encapsulated strains (R6, T1 and T2), and five clinical isolates belonging to two circulating pneumococcal clones, ST63^8^ (2 strains) and ST156^9V^ (3 strains). Within each group, there were strains with both low level of resistance (LL-R) to CIP (carrying mutations in *parC*), and strains with high-resistance (HL-R) to CIP (carrying mutations in both *parC* and *gyrA*). The breakpoints of the Clinical and Laboratory Standards Institute [30] were considered for LVX and MXF. Breakpoints for CIP were taken from our previous studies [26,27]. Strains were considered susceptible to SCN if their MICs were similar to strain R6 (8 μg/ml = 25 μM), based on a previous study [33]. The five strains LL-R to CIP were susceptible to LVX and MXF. The three strains HL-R to CIP were also highly resistant of LVX and MXF. However, all strains were susceptible to SCN.

**Fig 1:**
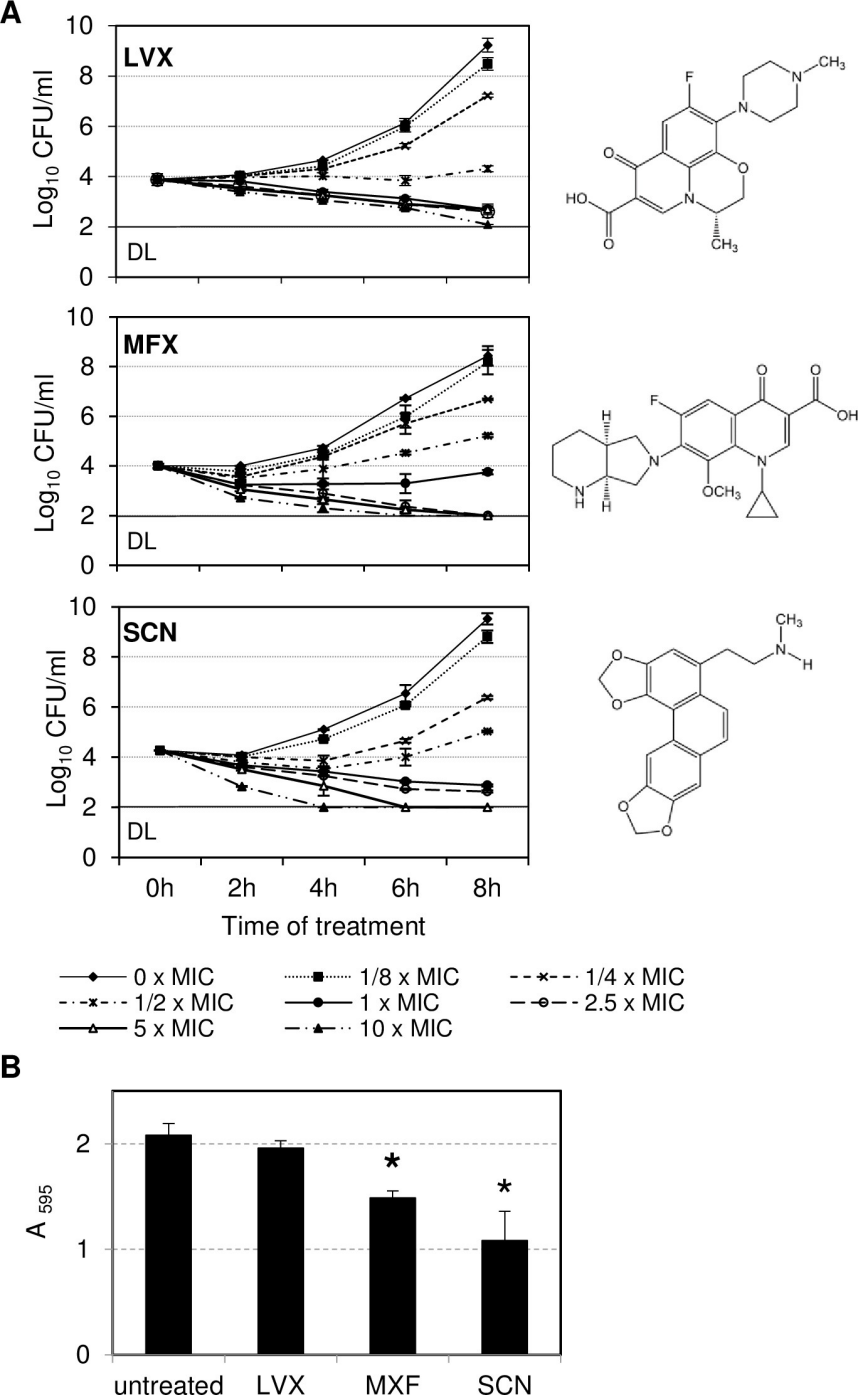
Bactericidal activity of drugs against strain *S*. *pneumoniae* R6. (A) Planktonic bacteria killing-curves in the absence or presence of the indicated drugs, which chemical structures are showed on the right. DL, detection limit (B) Inhibition of 6h-biofilm treated with 5 × MIC for 24 h, quantified by crystal violet staining. The results are the mean ± SD of two (A) and three (B) independent experiments. *, *P* < 0.05 versus untreated.

Killing-curves of planktonic bacteria (Fig 1A) demonstrated similar dead kinetics at supra- MIC concentrations with any of the three drugs. Decrease in CFUs was proportional to the concentration of drug used and to the duration of the treatment. MXF showed greater bactericidal activity than LVX, decreasing CFU to less than 2 Log_10_ at concentrations ≥ 2.5 × MIC after 6 h, while for LVX this decrease was only observed at higher concentrations (10 × MIC) after longer treatments (8 h). SCN showed the highest bactericidal activity, reducing viability of planktonic cells to less than 2 Log_10_ after 4 or 6 h of treatment with 10 × MIC or 5 × MIC, respectively. SCN and MXF, but not LVX, reduced also biomass of biofilm at 5 × MIC after 24 h of treatment (Fig 1B), being SCN the most effective drug.

### PAEs were longer in planktonic bacteria when the target of the treatment was Topo I, independently of the susceptibility to FQs

To analyze the influence of FQ-susceptibility in PAE induced by FQs or SCN, we used strains with similar genotypes but different amino acid changes associated to FQ-resistance. FQs induced a significant PAE at 10 × MIC in all strains (Table 2). However, at 2.5 × MIC no significant PAE either in the non-encapsulated Cip-resistant strains or in the HL-Cip-resistant R isolate of the ST63^8^ clone was induced. PAE induced by MXF were similar or longer than that induced by LVX. PAE induced by either of the two FQs was shorter in resistant strains than in susceptible strains. In contrast, SCN induced significant PAE in all strains at every concentration used and there were no differences between susceptible and FQ-resistant strains. In addition, SCN induced PAEs longer than that induced by LVX and/or MXF (Table 2).

**Table 2:** PAE in planktonic *S*. *pneumoniae* strains after 1 h exposure to drugs, measured by viable count method.

Group	Strain	PAE (h ± SD)^a^					
		LVX (× MIC)		MXF(× MIC)		SCN(× MIC)	
		2.5	10	2.5	10	2.5	10
Laboratory strains	R6	**1.02 ± 0.12**	**1.67 ± 0.06**	0.39 ± 0.12	**1.33 ± 0.29**	**1.00 ± 0.05**^**◻**^	**1.87 ± 0.12** ^**◻**^
	T1	0.40 ± 0.05 *	**0.70 ± 0.06** *	0.00 ± 0.00	**0.66 ± 0.05** *	**1.03 ± 0.06**^**♦◻**^	**1.95 ± 0.20** ^**♦◻**^
	T2	0.28 ± 0.05 *^•^	**0.56 ± 0.11** *	0.48 ± 0.07	**0.70 ± 0.11** *	**0.89 ± 0.21**^**♦◻**^	**1.98 ± 0.13** ^**♦◻**^
ST63^8^ clone	2390	**1.41 ± 0.21**	**1.68 ± 0.29**	**1.43 ± 0.35**	**2.56 ± 0.13**	**1.68 ± 0.29**	**2.22 ± 0.19** ^**♦**^
	3498	**0.57 ± 0.10** ^•^	**1.18 ± 0.23**	**1.10 ± 0.26**	**1.99 ± 0.16** ^•^	**1.77 ± 0.10** ^**♦◻**^	**2.22 ± 0.07** ^**♦**^
ST156^9V^ clone	3360	**1.22 ± 0.17**	**2.06 ± 0.35**	**0.90 ± 0.21**	**1.71 ± 0.31**	**1.52 ± 0.10** ^**◻**^	**1.76 ± 0.17**
	2194	**0.77 ± 0.10** *	1.09 ± 0.02*	**0.74 ± 0.04** *	**1.45 ± 0.13**	**1.60 ± 0.31** ^**♦◻**^	**1.88 ± 0.32** ^**♦◻**^
	1920	0.22 ± 0.05 *^•^	0.97 ± 0.07*	0.49 ± 0.12 *	**1.00 ± 0.15** *^•^	**1.62 ± 0.20** ^**♦◻**^	**1.72 ± 0.25** ^**♦◻**^

^a^ PAE values considered significant (≥ 0.5 h) are indicated in boldface. Results are the average ± SD of three independent replicates. Significant differences in PAE values (*P* < 0.05):

^**♦**^longer with SCN than with LVX at similar concentration in the same strain

^◻^longer with SCN than withMXF at similar concentration in the same strain

*shorter in the CipR strain than in the CipS strain of similar genotype

^**•**^ shorter in the HL-R strain than in the LL-CipR strain of similar genotype.

**Fig 2:**
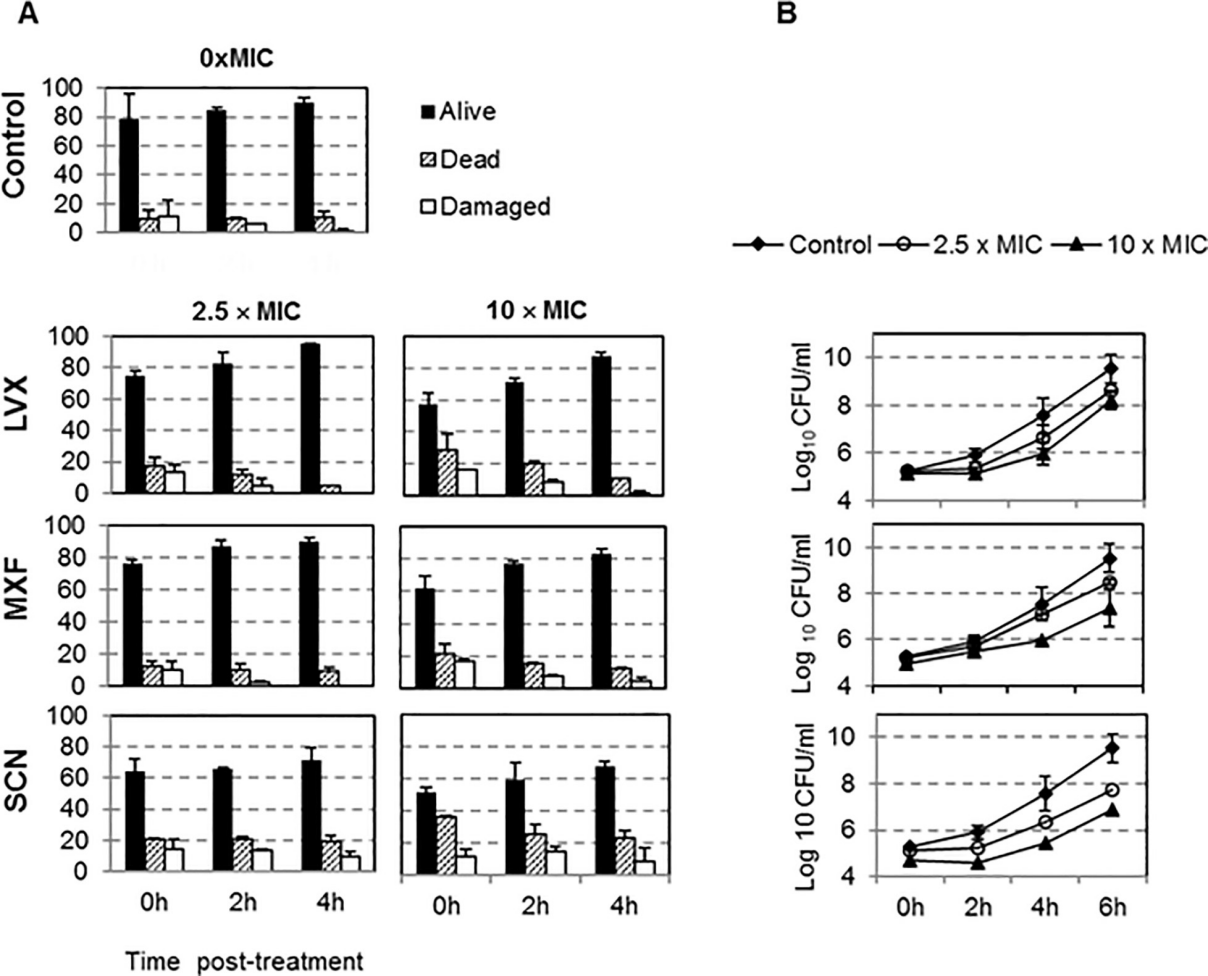
Viability of planktonic R6 in PAE-phase. Cultures containing 10^8^ CFU/ml were exposed for 1 h to the indicated drug concentrations, diluted 1000-fold in drug-free media and incubated during 6 h. (A) Quantification of alive (black bars), damaged (hatched bars), or dead (white bars) bacteria in PAE-phase stained with the BacLight kit and counting by fluorescence microscopy BXG1 (100 bacteria for each condition). (B) Growth kinetics of bacteria in PAE-phase. The results are the mean ± SD of two or three independent experiments.

The viability of planktonic bacteria in PAE phase was estimated for the R6 strain by fluorescence microscopy (Fig 2). Treatments of 1 h with 2.5−10 × MIC decreased living bacteria by up to 75–57%, 77–62% and 65–52%, for LVX, MXF and SCN, respectively, whereas in control cultures these values were of up to 80% (0 h in Fig 2). Four hours after finishing the treatment (4 h in Fig 2), an increase in live bacteria close to the control values was observed for the FQs, but not for SCN (74–68%). Therefore, SCN treatment was the most effective in reducing bacteria viability, not only when the antibiotic was present but also after removing it.

### SCN was more effective than FQs in reducing PAE, adherence and thickness of biofilms

PAE induced after 1 h of treatment with 1−5 × MIC of LVX, MXF or SCN in R6 sessile bacteria forming biofilms was determined. The three drugs induced a significant PAE in planktonic cells (Table 3). They also induced PAE in sessile bacteria forming biofilms, with the exception of MXF at 1 × MIC in the resistant strain T2. Although T2 and R6 demonstrated similar capacity to form biofilm (S1 Fig), PAE induced by FQs, but not by SCN, was shorter in the resistant T2 strain than in the susceptible R6 strain.

**Table 3:** PAE in sessile *S*. *pneumoniae* strains forming biofilms after 1 h exposure to drug, measured by viable count method.

Strain	PAE (h ± SD)^a^								
	LVX (× MIC)			MXF (× MIC)			SCN (× MIC)		
	1	2.5	5	1	2.5	5	1	2.5	5
R6	**0.99 ± 0.16**	**2.23 ± 0.10**	**3.32 ± 0.03**	**0.89 ± 0.18**	**1.20 ± 0.21**	**1.91 ± 0.24**	**0.84 ± 0.09**	**1.26 ± 0.08**	**2.31 ± 0.36**
T2	**1.21 ± 0.18**	1.57 ± 0.27*	1.89 ± 0.37*	0.25 ± 0.17*	0.76 ± 0.13*	1.34 ± 0.15*	**0.76 ± 0.12**^**◻**^	**1.53 ± 0.19**^**◻**^	**2.47 ± 0.07**^**◻♦**^

^a^ PAE values, considered significant (≥ 0.5 h) are indicated in boldface. Results are the average ± SD of three independent replicates. Significant differences in PAE values (*P* < 0.05)

^♦^ longer with SCN than with LVX at similar concentration in the same strain

^◻^ longer with SCN than with MXF at similar concentration in the same strain

* shorter in the HL-R strain T2 than in the S strain R6.

**Fig 3:**
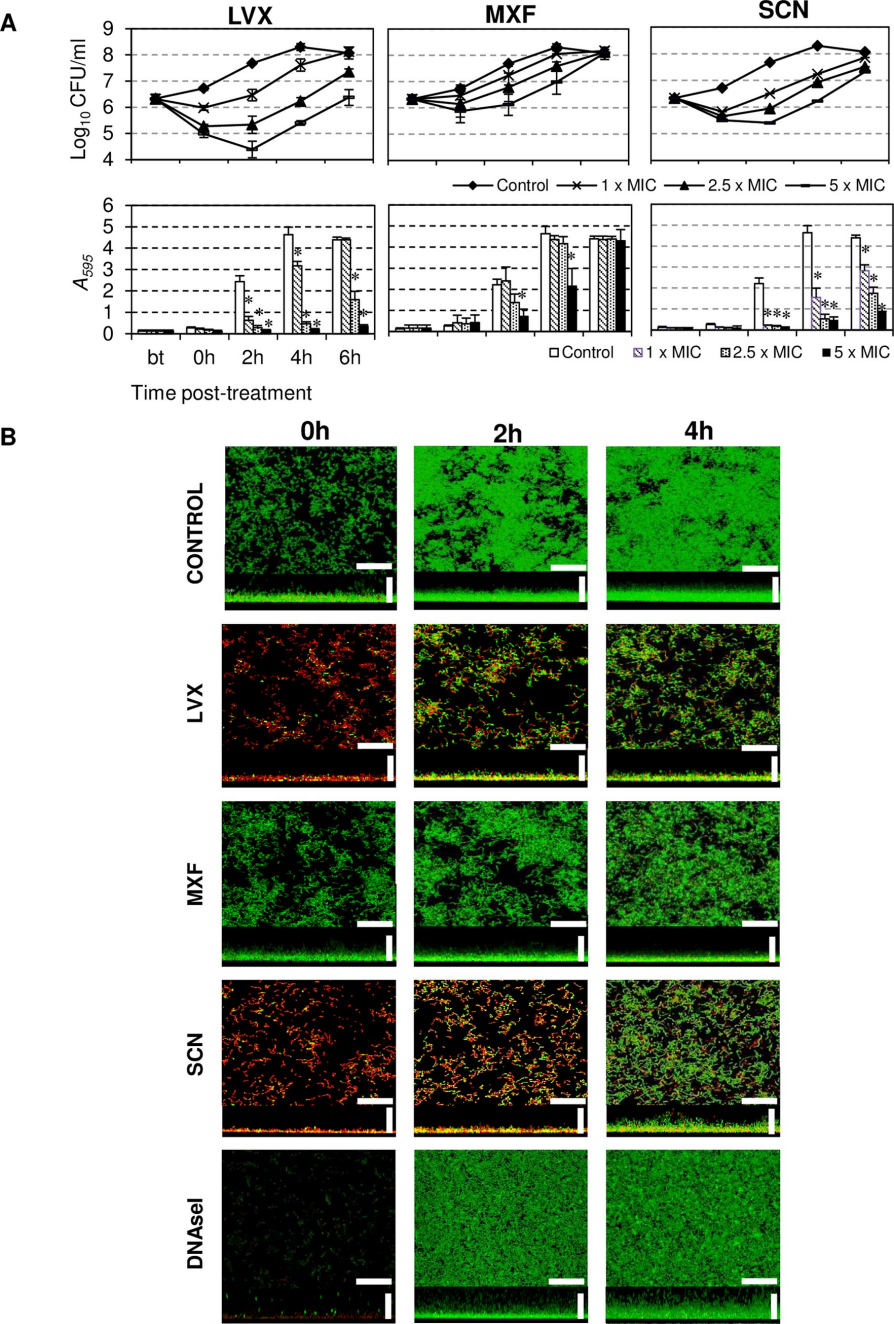
Biofilm formation by R6 in PAE-phase. About 10^6^ CFU/ml attached bacteria were treated for 1 h with the indicated drugs. Treatment was finished by washing and bacteria were incubated at 34°C for 6 h. (A) Growth kinetics of sessile bacteria forming the biofilm (viable counting) and quantification of bacteria in the biofilm (staining with crystal violet). The results are the mean ± SD of four independent experiments. *, *P* < 0.05 versus untreated. Bt, before treatment. (B) Confocal structure of the biofilm after treatment at 5 × MIC (confocal microscopy) and a control treated with DNAseI at 50 μg/ml, which has been showed to disrupt biofilms [44] Scale bars 25 μm.

PAE induced in sessile bacteria after treatment delayed their growth (viable counting in Fig 3A), and reduced biofilm formation (Fig 3A). The biofilm was thinner, the higher the concentration used in the treatment. PAE and biofilm reduction were greater after treatments with SCN or LVX than with MXF. Thus, 6 h after the end of the treatment with 1, 2.5, or 5 × MIC, A_595_ of biofilm remained 0, 2.7 or 10-fold lower than the untreated biofilm for LVX while it was 1.5, 2.5, or 5-fold lower for SCN (Fig 3A). However, only after treatment with ≥ 5 × MIC of MXF a reduction of the biofilm formation was observed and only at 4 h.

The evolution and architecture of biofilms formed by strain R6 in the PAE-phase were studied by confocal laser scanning microscope after drug treatment with 5 × MIC. Viability of bacteria in the biofilm was monitored using the *Bac*Light LIVE/DEAD bacterial viability kit (Fig 3B). Treatment of 1 h with either SCN or LVX, but not with MXF, killed most bacteria (0 h in Fig 3B), being the thickness of biofilms of 10.09 ± 0.79 μm (control), 7.18 ± 0.58 μm (LVX), 17.08 ± 1.02 μm (MXF) and 2.91 ± 0.43 μm (SCN). Therefore, the thickness of biofilms was lower under treatment with LVX (about 1.4-fold) or SCN (about 3.5-fold) than in the untreated control. Recovery of the biofilm was observed after 4 h of finishing the treatment with SCN or LVX (4h in Fig 3B). *S*. *pneumoniae* biofilm formation requires the presence of extracellular DNA as a component of the extracellular matrix, which is needed to establish and maintain pneumococcal biofilms [7]. After treatment of 1h with DNAaseI, the biofilm was practically eliminated. However, after 2 and 4h of finishing DNAaseI treatment, both biofilm and viable bacteria recovered (Fig 3B).

## Discussion

The environment influences different bacterial factors (virulence, motility, biofilm formation, antimicrobial susceptibility) by modulating the supercoiling of bacterial DNA [44]. The level of supercoiling in *S*. *pneumoniae* is regulated by the activity of their DNA topoisomerases, i.e., gyrase, Topo IV and Topo I [23]. Mutant gyrase and Topo IV enzymes, present in FQ-resistant isolates, have lower activities than wild-type enzymes, influencing the supercoiling level [45]. Therefore, to study the activity of the Topo I inhibitor SCN, it was necessary to consider both wild-type strains and isogenic derivatives carrying gyrase and Topo IV mutations (R6, T1 and T2). These strains are non-encapsulated. Clinical non-encapsulated pneumococci represent an emerging problem in pneumococcal infections. In addition, five clinical strains were also included. They belong to the two clones more closely related with FQ resistance in Spain in 2009, as a consequence of the introduction of PCV7, ST156^9V^ and ST63^8^ ☯26]. All strains studied were susceptible to SCN, regardless of their susceptibility to FQs.

Our data shows a higher bactericidal activity for MXF than for LVX in planktonic bacteria and in inhibition of 6h-biofilm after 24 h treatment. This is in accordance with previous studies showing greater *in vitro* activity [46], faster rates of killing [47], and more *in vivo* efficacy [48] of MXF compared to LVX or other fluoroquinolones [49,50] against planktonic *S*. *pneumoniae*. Likewise, MXF has been showed to be more effective than LVX in disrupting *S*. *pneumoniae* biofilms 6h-biofilm at concentrations achieved in the bronchial mucosa [51]. Another study reports a lower activity of LVX against pneumococcal biofilms than against planktonic cells [52], which supports our results. Other authors also reported that MXF reduced more than LVX the viability of 2 days-biofilm of R6 [53]. In any case, the compound that showed the highest and fastest bactericidal activity against planktonic and biofilms in our study was SNC. The bactericidal activity of other alkaloids against streptococci has been identified very recently, such as berberine against *Streptococcus agalactiae* [54] or alkaloids from seeds of the *Sterculia lychnophora* tree against *Streptococcus mutans* [55].

This study also established that LVX, MXF and SCN were able to induce PAE in planktonic bacteria and biofilms. PAE is an important pharmacodynamic parameter with a great clinical significance. For many compounds it has been demonstrated that prolonged *in vitro* PAEs may also suppress bacterial growth *in vivo* during periods of low drug concentration [56]. PAE needs to be considered in antibacterial dosing regimens, so that the greater the PAE induced by an antibiotic, the less frequently it should be administered. This will reduce toxicity, development of resistance, and treatment costs [57]. The clinical relevance of this effect will be even greater in biofilms, which need longer treatments. PAE is a well-established parameter for different kind of antibiotics against several bacteria on planktonic state. Some studies, which support our results, have reported that PAE induced by LVX [58] or MXF [59] in planktonic cultures of *S*. *pneumoniae*. We have recently studied the molecular mechanisms involved in this process and found that the production of reactive oxygen species, which is a consequence of transcriptional alterations induced by the drugs, is a major PAE contributor [60]. However, PAE has never been studied in biofilms. As far as we know, this is the first study that analyzes the biofilm formation capacity of *S*. *pneumonaie* surviving treatment. Interestingly, we observed that MXF induced PAE similar or longer than LVX in planktonic bacteria (exponential phase), but shorter in sessile bacteria forming biofilms (stationary phase), suggesting a relation between PAE and growth phase. These results agree with previous studies showing that the bactericidal activity of MXF was greater than that of LVX in the exponential growth phase, but the opposite was true in the stationary phase [61]. In the present study, MXF reduced biofilm formation more efficiently than LVX in treatments of 24 h, i.e., long enough not to be influenced by the growth phase. However, short treatments, used to induce PAE, were strongly influenced by the stationary phase of the biofilm, demonstrating that LVX has a higher bactericidal activity than MXF, causes a higher reduction in the viability of sessile pneumococci and induces longer PAE. Once again, SCN was more effective than FQs, inducing longer PAE in planktonic bacteria. SCN also induced longer PAE than MXF in biofilm. Moreover, SCN was the compound causing the highest reduction in the viability and adhesion of sessile bacteria, decreasing biofilm thickness and the size of monolayers. Recent studies in *Pseudomonas aeruginosa* support the relationship established in our study between TopoI and the formation of biofilms [62]. It has also been described the anti-biofilm effect of other alkaloid, based on the inhibition of effective adhesion and growth of bacteria forming the biofilm [63]. If adequate levels of SCN can be achieved *in vivo*, the low adherence after alkaloid treatment could imply a clinical advantage, since SCN could prevent or obstruct the chronicity of infections associated with the formation of biofilms [64].

In this study, we found an additional advantage of SCN compared to LVX and MXF. Both FQs, but not SCN, showed a significant decrease in its inhibitory activity against resistant strains and induced shorter PAE in them than in susceptible strains. As higher was the resistance to FQs, the shorter was the PAE induced by both FQs, decreasing up to 5-fold (LVX) or 2-fold (MXF) in planktonic cells, and up to 1.7 (LVX) or 1.4−fold (MXF) during biofilm formation. These results confirmed similar observations in other gram-positive cocci. PAE induced by LVX and gatifloxacin in FQ-sensitive *Staphylococcus aureus* strains were shorter than those observed in resistant strains [65]. For vancomycin-resistant strains of *Enterococcus faecium*, the PAEs were considerably shorter than those for vancomycin-susceptible strains [66].

Summarizing, Topo I is an appropriate antimicrobial target in *S*. *pneumoniae*, especially in strains resistant to inhibitors of other topoisomerases (FQs). TopoI inhibitors such as SCN showed faster and higher bactericidal activity than FQs regardless of growth phase; induced longer PAEs in planktonic bacteria, reduced more than FQs the thickness of mature biofilm and interfered with biofilm formation. In addition, TopoI inhibitor activity did not decrease in FQ-resistant strains. All these results support Topo I inhibitors as new therapeutic candidates for the treatment of pneumococcal diseases and for the prevention of the chronicity of the pneumococcal disease.

## Supporting information

S1 Fig Biofilm formation capacity of R6 and T2. **S**trains were grown in CpH8 to OD_595_ = 0.5, diluted 1/100 and dispensed in 96-well flat-bottom polystyrene microtiter dishes. Plates were incubated at 34°C to get 10^6^ CFU/ml of cultivable bacteria adhered to the walls base. Then, attached bacteria were rinsed three times with CpH8 and incubated for 6 h to analyze biofilm formation on polystyrene plates. Growth in biofilm was quantified by Absorbance and viable count method.
(TIF)

S1 File (XLSX)
