# Supplementary figures and images for: Antibacterial activity of a DNA topoisomerase I inhibitor versus fluoroquinolones in Streptococcus pneumoniae
Source: PLoS One. 2020 Nov 3;15(11):e0241780. doi: 10.1371/journal.pone.0241780 (PMC7608930; doi:10.1371/journal.pone.0241780)

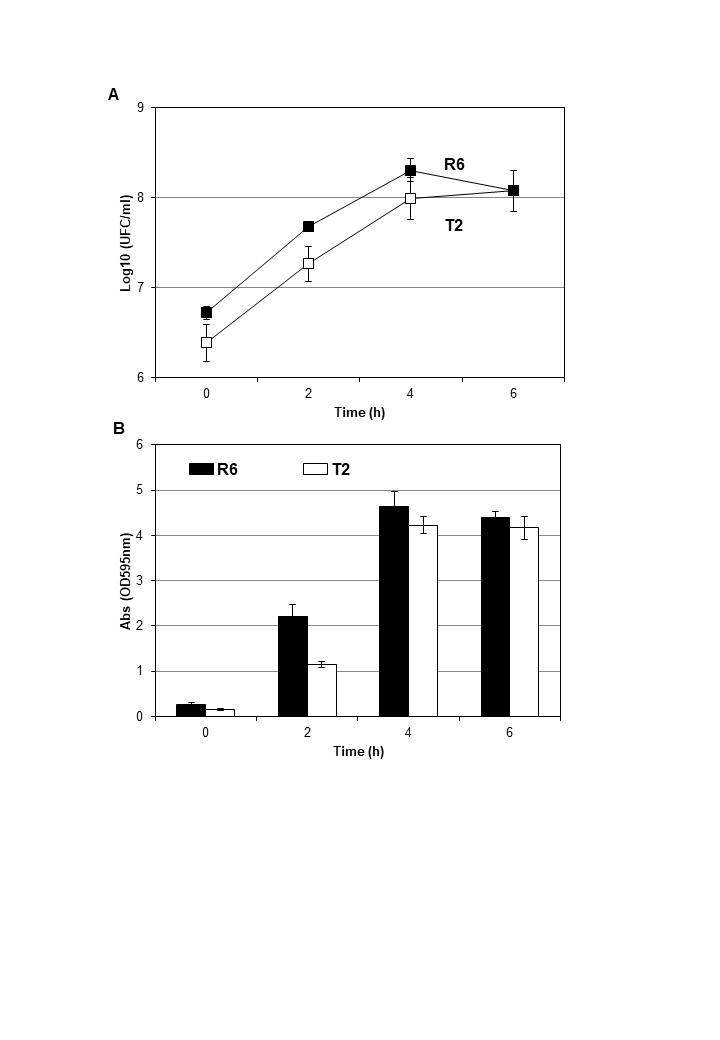

Supplement: S1 Fig — Strains were grown in CpH8 to OD595 = 0.5, diluted 1/100 and dispensed in 96-well flat-bottom polystyrene microtiter dishes. Plates were incubated at 34°C to get 106 CFU/ml of cultivable bacteria adhered to the walls base. Then, attached bacteria were rinsed three times with CpH8 and incubated for 6 h to analyze biofilm formation on polystyrene plates. Growth in biofilm was quantified by Absorbance and viable count method. (TIF) [file pone.0241780.s001.tif]
